# Musculoskeletal anatomy education perspectives

**DOI:** 10.1007/s00296-026-06175-4

**Published:** 2026-06-04

**Authors:** Uliana Pidvalna, Lesya Mateshuk-Vatseba, Baimakhan Tanabayev, Ahmet Usen

**Affiliations:** 1 Department of Normal Anatomy, Danylo Halytsky Lviv National Medical University, Lviv, Ukraine; 2 Ukrainian-Polish Heart Center Lviv, Lviv, Ukraine; 3 Department of Morphophysiology, South Kazakhstan Medical Academy, Shymkent, Kazakhstan; 4 Department of Physical Medicine and Rehabilitation, Medipol University Faculty of Medicine, TEM Avrupa Otoyolu Göztepe Çıkışı No: 1, Bagcilar, 34214 Istanbul, Türkiye

**Keywords:** Musculoskeletal system, Anatomy, Education, Curriculum

## Abstract

Musculoskeletal (MSK) anatomy education is a critical foundation for developing competency among radiologists, physiatrists, rheumatologists, and orthopedic surgeons. However, current undergraduate medical curricula often exhibit significant deficiencies in instructional hours, integration of diverse teaching modalities, and clinical relevance. This narrative review synthesizes recent evidence (March 2021–March 2026) identified through a targeted search of Medline, Embase, and Scopus, with an emphasis on consensus guidelines, validation studies, and clinically focused publications related to MSK anatomy, imaging modalities (ultrasound, magnetic resonance imaging, computed tomography), and curriculum design for medical students in relevant specialties. Multimodal interventions, including cadaveric dissection, radiological anatomy, case-based rheumatologic and rehabilitation modules, and technology-enhanced platforms such as 3D virtual models and AI-driven adaptive learning, have been associated with improvements in knowledge retention, spatial reasoning, diagnostic accuracy, and procedural confidence compared with didactic instruction alone. Persistent knowledge gaps undermine interpretive proficiency in MSK imaging, including the identification of synovitis and enthesopathy, and are linked to reduced clinical preparedness. Objective assessments reveal suboptimal performance despite completion of conventional preclinical training. Cadaveric dissection fosters practical skills and ethical professionalism, while early integration of imaging connects theoretical morphology with three-dimensional relational understanding and pathological correlations in MSK and rheumatic diseases. Implementation frameworks recommend phased rollouts that incorporate stakeholder needs assessments, faculty development through train-the-trainer models, resource reallocation for point-of-care ultrasound and virtual reality, and strategies to address barriers, such as grants for low-resource settings and modularization to reduce curricular congestion. These evidence-based approaches support scalable reforms that produce MSK-literate clinicians prepared for precision diagnostics and interventional practice.

## Introduction

Musculoskeletal (MSK) anatomy education is central to the training of radiologists, physiatrists, rheumatologists, and orthopedic surgeons. For future radiologists, precise knowledge of MSK anatomy underpins accurate interpretation of magnetic resonance imaging (MRI), computed tomography (CT), and ultrasound (US) of ligaments, tendons, cartilage, and bone marrow [1]. For physiatrists and rheumatologists, integrating anatomy with physical examination and imaging enables early detection of inflammatory synovitis, tenosynovitis, and enthesitis, and guides targeted injections [2]. Orthopedic surgeons rely on detailed segmental and regional anatomy for safe approaches, fracture fixation, and joint-preserving procedures. These specialists should interpret imaging, perform injections, differentiate mechanical from inflammatory pathology, and communicate with patients and other surgeons about joint and soft-tissue structures. Meanwhile, many international curricula remain under-resourced in MSK content [3, 4].

Reports from the USA and Europe indicate that many undergraduate and graduate programs allocate too few hours to MSK topics, leading to poor clinical performance and diagnostic confidence [3, 5, 6]. Some recent undergraduate reforms recommend roughly 40–60 h of dedicated MSK instruction during the preclinical phase, yet many medical schools still fall markedly short of this [3].

This raises the question of whether current curricula for MSK anatomy education meet the modern needs of MSK knowledge for medical students and residents in future clinical practice for radiologists, physiatrists, rheumatologists, and orthopedic surgeons.

This narrative review aims to map current evidence and practical perspectives on upgrading MSK anatomy curricula for medical students and residents, with an emphasis on didactic, imaging, and clinical integration, as well as technology-enhanced curricula to bridge anatomy and practice.

## Search strategy

We conducted a narrative review to summarize the current state and practical considerations of MSK anatomy education. The search strategy was developed based on the recommendations proposed by Gasparyan et al. [7]. A structured literature search was performed in Medline/PubMed, Embase, and Scopus, using terms centered on “musculoskeletal anatomy,” “MSK anatomy education,” “MSK ultrasound,” “MSK MRI,” “musculoskeletal curriculum,” and specialty-specific strings (rheumatology, physiatry, radiology, orthopedic surgery). We analyzed English articles only. Preference was given to the recently published documents. Priority was given to consensus documents, validation studies, and clinically oriented reviews applicable to MSK practice. Manual backward citation chasing was used to identify key older references on curriculum design and anatomy teaching. Reference lists of key articles were manually screened to identify additional relevant publications.

### Insufficient musculoskeletal anatomy competency across training levels

Published studies consistently report insufficient competency in MSK anatomy among medical trainees and early-career physicians. Objective assessments demonstrated that knowledge gaps persist despite completion of standard preclinical anatomy curricula [4, 8, 9]. Meanwhile, practitioners maintained their MSK knowledge, scoring higher than the undergraduate group but not significantly different from the postgraduate group. A study suggests that MSK anatomical knowledge was retained after university graduation, supporting the idea of improving medical curricula for students rather than postponing it until graduation [10]. Evidence of widespread gaps in musculoskeletal anatomy education across training levels is presented in Table 1.

**Table 1:** Evidence of competency gaps in musculoskeletal (MSK) anatomy education

Domain	Study	Key evidence from the literature	Educational implication
Undergraduate MSK knowledge	Harkins et al. [4]; Peeler [8]	Persistent deficits despite completion of standard anatomy curricula	Current MSK teaching is insufficient in depth and/or duration
Knowledge retention after graduation	Olufade et al. [9]; Giuriato et al. [10]	Graduates retain MSK knowledge better than undergraduates, but still show suboptimal performance	Earlier and longitudinal integration may improve retention
Clinical confidence (MSK examination)	Sonnleitner et al. [2]; Qazi et al. [11]	Consistently lower confidence in MSK physical exam vs. other systems	MSK clinical skills training is underdeveloped
Imaging interpretation performance	Factor et al. [12]	Trainees demonstrate reduced accuracy in MSK imaging interpretation	Weak integration between anatomy teaching and radiological application
Curriculum structure surveys	Mushtaq et al. [1]; Orchard et al. [3]	Limited dedicated MSK teaching hours in many medical programs	Structural underrepresentation of MSK content is widespread

The educational modality appeared to influence outcomes significantly. Programs that incorporated multimodal teaching strategies, such as cadaveric dissection combined with imaging-based learning and clinical case discussions, reported higher assessment scores than those relying primarily on didactic lectures or prosection alone. In particular, integrating radiological anatomy improved learners’ ability to translate theoretical knowledge into clinical contexts [3]. A range of educational modalities has been evaluated for improving musculoskeletal anatomy learning outcomes, with varying degrees of effectiveness (Table 2).

**Table 2:** Educational modalities in MSK anatomy teaching and reported outcomes

Modality	Study	Examples of implementation	Reported outcomes	Educational value
Cadaveric dissection	Blake et al. [13]; Baratz et al. [14]	Full dissection, prosection-based labs	Improved spatial understanding, anatomical retention, professionalism	High impact for improving practical skills and structural mastery
Radiology-integrated anatomy	Becker [15]; Madison et al. [16]	MRI, CT, ultrasound correlation with anatomy teaching	Improved translation of anatomy to imaging interpretation	Strong clinical correlation and diagnostic reasoning; high impact for 3D anatomy
Case-based learning	Bassey et al. [17]	Rheumatology, orthopedics, and rehabilitation case modules	Enhanced clinical reasoning and pathology integration	High value for applied clinical thinking
MSK ultrasound training	Huang et al. [18]	Joint scanning, dynamic tendon assessment, injection guidance, and anatomy	Improved procedural confidence and bedside anatomy understanding	Essential for modern point-of-care skills
Technology-enhanced learning	Zayachkivska [19]; Gladman et al. [20]; Velez-Martinez et al. [21]; Beaulieu et al. [22]	3D anatomy platforms, AI-driven adaptive systems	Increased engagement and individualized learning pathways	Supports flexible and self-directed learning
Supplemental instruction	Wilson et al. [23]	Peer-assisted learning, tutor-led reinforcement sessions	Improved exam performance and learner confidence	Effective for knowledge consolidation
Intensive short courses	Peeler [24]; Mushtaq et al. [25]; Bandekar et al. [26]	2-week focused MSK modules	High student satisfaction and rapid knowledge gain	Efficient reinforcement strategy

#### Anatomical education and cadaver-training

Dissection still plays a key role in MSK education, especially at the undergraduate level. Nothing will replace the proper dissection classes [27]. Working with cadavers, students can learn not only morphological anatomy but also the ethical considerations involved in working with the human body. Studies of student preferences and proficiency show that proctor-assisted cadaver dissection is among the most effective and preferred methods for learning MSK anatomy, particularly for complex 3D relationships. Dissection training develops manual dexterity, spatial reasoning, variant identification, teamwork, and professional attitudes toward the body, all of which are relevant to both surgeons and interventional non-surgeons [27]. The supplemental instruction improves student outcomes, including greater perceived readiness for anatomy assessments and alignment with preferred learning styles in undergraduate human anatomy curricula [23]. Traditional materials remain important and should be supplemented, not replaced. However, in many postgraduate programs, targeted prosections or dissection-style stations (rather than full-body dissection) are more practical and can be combined with imaging and simulation.

Generally, anatomical dissection classes are followed by printed outlines and textbooks/atlases, such as Classic Anatomy: *Gray’s Anatomy* (sectional or clinical editions), *Netter’s Atlas of Human Anatomy*,* Clinical Anatomy by Regions* [28], *Thieme’s Atlas of Anatomy*, etc. Surely, for MSK-focused dissection materials, such as Orthopedic Physical Assessment (Magee, 2008), Clinical Anatomy in Action (Palastanga et al., 1994), and specialty MSK-rheumatology/MSK-radiology atlases, are often adopted in postgraduate workshops. However, is it enough for modern medicine? 

With rapid advances in radiology, we need to adapt, and one option is to include radiological anatomy in the medical student curriculum, ideally from the first year. The most up-to-date are published articles in peer-reviewed journals. Limitations to non-open-access journals, especially in low- and middle-income countries, widen the gap in sharpening skills. The European Society of Radiology’s (ESR) initiative to develop the ESR e-Book as an open-access digital teaching resource, including an MSK chapter that aligns with the undergraduate European Training Curriculum for Radiology, seems especially useful [15].

The mean time spent teaching musculoskeletal anatomy varies across countries, with programs that include didactic lectures, case-based learning, workshops, and small-group tutorials [4, 8, 9]. However, the majority report “lack of confidence in the MSK physical exam, which was notably lower than in other organ system exams” [2, 3, 29]. It draws our attention to questions about how to improve the curriculum to meet modern clinical needs and students’ expectations.

Theoretical knowledge is the basis, but without proper practice, it would not be applied in clinical practice. For first-year medical students, this typically begins with dissection courses. Appropriate MSK anatomy laboratory/cadaveric sessions are designed to align MSK anatomical theory with topographical anatomy during the first years. Traditional cadaveric dissection and prosection remain highly rated by students for learning complex 3D relationships and spatial reasoning, especially in the MSK system.

Imaging-based anatomy is now core to postgraduate training for rheumatologists, orthopedic surgeons, physiatrists, and even family doctors [29]. Recent publications suggest that “physician training in the area of MSK medicine has historically been inadequate,” and future doctors are not satisfied with their MSK knowledge [8]. Such a situation is typical for many countries [3, 5, 30]. The perspective in MSK anatomy education relies on upgrading curricula for medical students and residents by implementing practical courses [9, 24, 31–33].

These perspectives might include radiology-based anatomy with essential techniques: (1) Musculoskeletal ultrasound (MSUS) with longitudinal and transverse scanning of major joints (shoulder, elbow, wrist/hand, hip, knee, ankle/foot); dynamic assessment of tendons, ligaments, and soft tissues using movement and stress maneuvers; injection-guidance anatomy (e.g., subacromial, glenohumeral, hip, knee, entheses). (2) MRI: emphasis on multiplanar imaging (coronal, sagittal, axial) of joints, cartilage, menisci, ligaments, and bone marrow; MSK-focused rheumatology blocks. (3) CT: valuable for complex fractures, spinal anatomy, and osseous tumors; best taught alongside orthopedic or trauma rotations.

A spiral curriculum could start with a basic anatomy and dissection course and later include imaging anatomy, with future clinical-pathology correlation across several rotations during residency. Such a proposal allows learners to “see anatomy in practice,” linking static dissection with clinical signs and pathology. Early MSK radiological anatomy education, such as ultrasound, has been successfully integrated into a large medical school program, with benefits for clinical education [34]. Mini-residency training program in MRI utilization in MSK is a good option with successful educational intervention [35]. The duration and timing can be adjusted to the University schedules and possibilities. Interestingly, the two-week MSK anatomy course had the highest overall satisfaction among all first-year anatomy courses [24]. In any case, the longitudinal repetition of skills, anatomy refreshment, and the inclusion of specific frameworks for MSK patient care are vitally important [31].

#### Clinical rheumatology and rehabilitation: focus themes

MSK anatomical curricula should be integrated with clinically relevant rheumatologic and rehabilitation syndromes to organize instruction around the tight integration of anatomy, imaging, and clinical correlation. In rheumatoid arthritis, particularly involving the hands and feet, the detailed anatomy of the metacarpophalangeal, proximal interphalangeal, wrist, and metatarsophalangeal joints is directly linked to disease-specific pathology. Emphasis on the synovium, joint capsules, periarticular ligaments, and flexor–extensor tendon systems may enable learners to correlate structural inflammation with imaging findings such as synovitis, tenosynovitis, and erosions. This integrated approach supports more accurate imaging interpretation and a better understanding of functional impairment.

Similarly, axial spondyloarthritis modules that incorporate sacroiliac joint and spinal anatomy alongside imaging features of sacroiliitis may improve recognition of early inflammatory changes. Teaching that highlights ligamentous structures, joint biomechanics, and their radiologic correlates may enhance the ability to connect anatomical disruption with clinical symptoms such as pain and stiffness.

Tendon- and enthesis-related conditions represent another domain. Focused instruction on structures such as the Achilles tendon, plantar fascia, rotator cuff, and patellar tendon, combined with ultrasound and MRI correlation, may improve learners’ ability to identify enthesitis and overuse injuries. These gains are particularly relevant, as ultrasound is a widely available, inexpensive, and readily accessible tool.

In osteoarthritis of the knee and hip, case-based modules linking articular cartilage, menisci, labrum, and periarticular stabilizers with degenerative imaging findings may improve diagnostic reasoning. This approach integrates the ability to relate structural degeneration to biomechanical dysfunction and clinical presentation.

Integrating these focus themes into competency-based training frameworks provides a structured pathway to achieving clearly defined learning outcomes and aligns with broader educational trends [36, 37]. Within this framework, incorporating imaging-centered instruction plays a critical role in addressing documented disparities in diagnostic accuracy across specialties and training levels, reinforcing the need for systematic, longitudinal exposure to MSK imaging [12]. Emerging evidence indicates that expanding participation in MSK image interpretation may enhance healthcare delivery; however, such expansion is contingent upon the development of targeted educational programs, formal training pathways, and appropriate curriculum support [30].

Overall, the results indicate that clinically oriented, case-based, and imaging-anchored thematic modules in rheumatology and rehabilitation significantly enhance the application of anatomical knowledge. These approaches enable trainees to “think anatomically” during both bedside evaluation and imaging interpretation, addressing current gaps in MSK education and improving readiness for clinical practice.

#### Implementation strategies and barriers

This section outlines a pragmatic roadmap, drawing on successful reforms, such as anatomy integration pilots of the American Association of Medical Colleges (AAMC). By addressing logistics upfront, institutions can achieve measurable improvements in MSK education without overhauling entire programs.

The literature frequently characterizes a stepwise rollout as a phased approach that begins on a limited scale to build momentum. The initial phase generally consists of a needs assessment conducted via anonymous online surveys and focus groups with students, residents, faculty, and clinicians, using key questions: “Rate your confidence in MSK anatomy on a 1–10 scale” and “What tech/tools would enhance learning?” to analyze data, prioritize gaps (e.g., imaging skills), and secure administrative support.

Pilot programs that include faculty development are typically implemented within a single cohort (e.g., 2-year students and residents). 3D software and virtual dissection platforms (e.g., 3D virtual anatomy platforms) are increasingly used as adjuncts and, when cadavers are limited, as substitutes. Resource allocation is generally managed through a structured line-item budget that includes initial investments in virtual reality headsets, software licenses, and point-of-care ultrasound (POCUS) devices.

The integration of accessible digital learning tools is extensively documented in MSK education. These tools encompass video-sharing platforms such as YouTube, which offer concise tutorials on MSK anatomy and imaging modalities, including MRI and ultrasound, as well as AI-driven applications that generate interactive quizzes. Furthermore, advanced 3D anatomical visualization tools based on imaging data provide adaptive learning pathways tailored to individual learner needs. Generally, sustainability is achieved by aligning with accreditation standards, integrating innovations into formal curricula, and systematically mapping educational interventions to established competency frameworks.

**Fig. 1:**
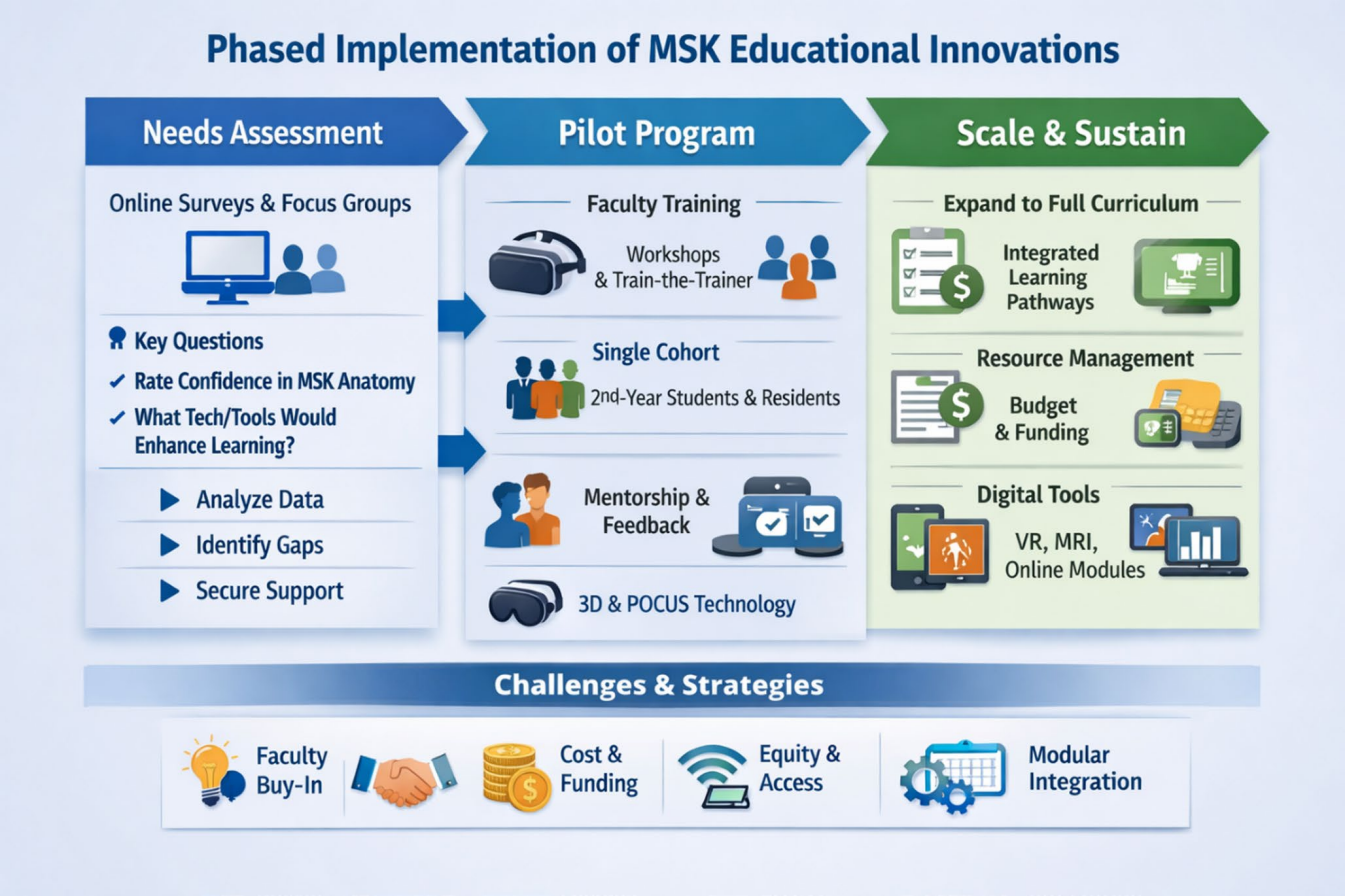
Phased implementation of musculoskeletal (MSK) educational innovations. The diagram illustrates a structured rollout model encompassing three sequential phases: needs assessment, pilot program, scale and sustain, supported by faculty training, digital learning tools, and evaluation strategies. Challenges and mitigation approaches are summarized at the bottom to highlight sustainability and equity considerations

Potential barriers may challenge the implementation of educational reforms. However, several proactive strategies may mitigate their impact. Faculty resistance may arise, particularly among experienced anatomists who prefer traditional teaching approaches, and this can be addressed by introducing appropriate incentives and recognizing educational innovation. Cost constraints may also limit adoption, especially in resource-limited settings; this can be mitigated by pursuing external funding sources, including competitive grants and public–private partnerships with industry stakeholders. Equity issues represent another important concern, as not all learners have access to personal devices or reliable high-speed internet, which may be partially resolved by prioritizing open-access educational tools and ensuring extended availability of well-equipped campus-based learning facilities. Time pressures within already densely structured curricula may also hinder integration, and these can be addressed by modularizing content and embedding it within existing clinical rotations to improve efficiency without increasing curricular load. Finally, evaluation gaps may limit the ability to demonstrate educational impact, requiring rigorous mixed-methods approaches that combine quantitative performance metrics with qualitative feedback from learners and faculty. A schematic overview of the phased implementation process is presented in Fig. 1, depicting the sequential stages and associated strategies for MSK curriculum reform.

Fig. 1 was created with the assistance of Microsoft Copilot. The authors reviewed and approved the final version of the image for accuracy and suitability in the context of this article.

Global inconsistencies in musculoskeletal (MSK) training underscore the urgent need for standardized, high-quality reforms to address curriculum gaps and safeguard educational integrity [32]. By proactively anticipating implementation barriers and leveraging evidence-based strategies, academic institutions may transform aspirational upgrades into achievable realities [20]. This narrative review demonstrates how supplemental instruction and iterative innovations not only boost perceived preparedness and learning preferences but also cultivate a new generation of MSK-proficient clinicians [28, 38].

## Conclusions

Inadequacies in current MSK anatomy curricula may translate into reduced diagnostic and procedural efficacy across radiology, physiatry, rheumatology, and orthopedic surgery. Longitudinal, competency-aligned upgrades to MSK anatomy instruction, beginning in the earliest university years and pairing cadaveric dissection with imaging-centric and clinically thematic instruction may result in knowledge retention, MSK-focused anatomical knowledge, and patient-centered application later on.

Pragmatic dissemination through needs-driven pilots, faculty upskilling, open-access digital repositories, and equitable technological adjuncts (e.g., virtual reality, AI-driven tools) may help in circumventing entrenched impediments, including fiscal constraints and time constraints. Institutional adoption of additional MSK-focused anatomical classes is the way to improve the level of MSK practitioners and thereby optimize diagnostic yield and therapeutic precision.

## Declarations

### Disclaimer

No part of this review is copied or published elsewhere in whole or in part. AI was not employed for writing and editing this review.
